# Assisted Synthesis of Coated Iron Oxide Nanoparticles for Magnetic Hyperthermia

**DOI:** 10.3390/nano12111870

**Published:** 2022-05-30

**Authors:** Liliana P. Ferreira, César P. Reis, Tiago T. Robalo, M. E. Melo Jorge, Paula Ferreira, Joana Gonçalves, Abdollah Hajalilou, Maria Margarida Cruz

**Affiliations:** 1 Department of Physics, University of Coimbra, 3004-516 Coimbra, Portugal; lpferreira@uc.pt; 2 Biosystems and Integrative Sciences Institute (BioISI), Faculdade de Ciências, Universidade de Lisboa, 1749-016 Lisboa, Portugal; cesar_reis93@hotmail.com (C.P.R.); ntrobalo@fc.ul.pt (T.T.R.); mdjorge@fc.ul.pt (M.E.M.J.); e.hajalilou@yahoo.com (A.H.); 3 CICECO-Aveiro Institute of Materials, Department of Materials and Ceramic Engineering, University of Aveiro, 3810-193 Aveiro, Portugal; pcferreira@ua.pt (P.F.); joanadfgoncalves@ua.pt (J.G.)

**Keywords:** magnetic hyperthermia, iron oxide nanoparticles, green synthesis, biocompatible coating

## Abstract

Magnetite nanoparticles were synthesized by the co-precipitation method with and without the assistance of an additive, namely, gelatin, agar-agar or pectin, using eco-friendly conditions and materials embodying a green synthesis process. X-ray diffraction and transmission electron microscopy were used to analyze the structure and morphology of the nanoparticles. Magnetic properties were investigated by SQUID magnetometry and ^57^Fe Mössbauer spectroscopy. The results show that the presence of the additives implies a higher reproducibility of the morphological magnetic nanoparticle characteristics compared with synthesis without any additive, with small differences associated with different additives. To assess their potential for magnetic hyperthermia, water-based suspensions of these nanoparticles were prepared with and without citric acid. The stable solutions obtained were studied for their structural, magnetic and heating efficiency properties. The results indicate that the best additive for the stabilization of a water-based emulsion and better heating efficiency is pectin or a combination of pectin and agar-agar, attaining an intrinsic loss power of 3.6 nWg^−1^.

## 1. Introduction

Nanostructured magnetic materials are currently an important theranostic tool in biomedical applications, including controlled drug delivery, magnetic resonance imaging (MRI), magnetic particle imaging (MPI) and magnetic fluid hyperthermia (MFH) [1,2,3,4,5,6]. Magneto-responsive systems are typically based on functionalized superparamagnetic iron oxide nanoparticles (SPIONs)—mainly Fe_3_O_4_ (magnetite) or γ-Fe_2_O_3_ (maghemite)—with sizes ranging between 5 and 20 nm [7,8]. At this moment, these oxides still represent the best compromise between magnetic properties, biocompatibility and toxicity for clinical application [5,9,10].

SPIONs are a key element in MFH, whereby a localized increase in temperature leads to an improved response to combined therapeutic approaches or to stand-alone cell thermoablation. This increase is due to energy dissipation by localized magnetic nanoparticles (MNPs) under an AC magnetic field through hysteresis losses and Néel or Brown relaxation. Although primarily envisaged for cancer therapy, MFH application may extend to other health-related issues (e.g., viral infections [11]).

MFH’s widespread use in a clinical environment still requires further development of suitable MNPs, particularly concerning their heating efficiency, since it would ensure effective treatment at lower MNP concentrations, thus avoiding toxicity problems. Attaining high values of heating efficiency is not straightforward, demanding a significant improvement of the routes leading to reproducible MNP distributions. Derived suspensions of suitable MNPs must also exhibit superior stability in the envisaged biological media, i.e., essentially water-based. Colloidal stability is dictated by particle size, surface chemistry and surface charge [12,13]. Thus, it is highly dependent on the coating layer, as well as on the non-polar or polar character of the suspension medium. A good choice of coating materials can also ensure long-term stability and tolerance whilst safeguarding the desired magnetic properties [8,12,13,14].

There are many routes to produce MNP: from standard co-precipitation (COP) to magnetic field- or protein-assisted synthesis [9,15,16,17]. Co-precipitation offers the advantages of high yield, low temperatures and easier scale-up, making it a particularly interesting process for industrial applications. However, COP typically lacks the necessary particle size and shape control that other methods can offer [18]. For magnetite in particular, broad size distributions have already been linked to a complex set of pathways leading to its formation [19]. Since MNP size and size distribution are crucial parameters for the envisaged application, overcoming this disadvantage would be an important step to re-position COP as a go-to MNP synthesis route. In addition, past years have seen a push towards environmentally friendlier chemistry through the use of biopolymers as capping agents [20,21], the use of natural fibers as templates [22] and the use of polycarboxylic acids for increased colloidal stability (e.g., citric acid) [23,24]. COP also enables the use of eco-friendly conditions and materials, well aligned with green chemistry principles [20].

Several attempts have been made to improve the size, size distribution and magnetic properties of co-precipitated Fe-based MNPs using readily available, biodegradable capping/stabilizing agents. Pectin, alginate, starch and chitosan are commonly found in the literature [21,25,26,27]. In addition to their role in the actual MNP synthesis, the presence of such polymers allows for direct coating. Due to steric repulsion, coating MNPs prevents surface-to-surface contact and irreversible clustering of the magnetic particles. Gelatin, pectin and agar-agar are also extensively used in the food and pharmaceutical industries, readily available, relatively cheap, non-toxic and present carboxyl, hydroxyl and/or amino groups, which are potential sites for conjugation and further chemical modification. Alves et al. [25] obtained promising results with gelatin-assisted COP synthesis of magnetite nanoparticles (NPs), namely, in the reduction of the nanoparticle size dispersion. Additionally, the authors reported improved magnetic properties when compared to agar-assisted and other studied methodologies (e.g., thermal decomposition). However, their water-based suspensions offered very limited stability since the corresponding suspensions were almost fully deposited on a time scale between one hour and one day.

Another important aspect for the envisaged application is the attainment of stable colloidal suspensions of superparamagnetic MNPs (ferrofluids). NPs tend to aggregate to minimize total surface energy, and in MNPs, the phenomenon is further exacerbated by the inter-particle magnetic dipolar attraction, with a further push towards destabilization. If these suspensions are to be stabilized, sufficient repulsive interactions must exist between particles, for which suitable surface functionalization, the careful choice of solvent and pH are paramount [28,29]. Water-based ferrofluids offer many advantages for biomedical applications but are often less stable and carry a lower MNP load than their non-polar counterparts [30]. Engineering MNPs into exhibiting hydrophilic behavior is the route to stable water-based dispersions and is typically achieved with suitable coating layers or the use of polar molecules as surfactants.

Citric acid (CA) with both carboxyl and hydroxyl functional groups, widely accepted as a stabilization agent in water-based ferrofluids, is commonly used as a coating layer prior to MNP dispersion [23,28,31,32]. Alternative methods for stabilization involve MNP dispersion in an aqueous CA solution, followed by temperature/pH-controlled adsorption and suspension steps [30,32]. There are suggestions that CA adsorbs on the surface of the magnetite NP by coordinating via one or two of its carboxylate groups, depending on the steric necessity and NP surface curvature, with at least one carboxylic acid group exposed to the solvent and responsible for the surface charge [28]. With a low pKa1 value (3.13), citric acid coatings are expected to be largely deprotonated in a broad pH range, including physiological pH. Chemisorption experiments showed that CA will lead to the monolayer coverage of the MNP, mostly with bidentate type surface complexes between carboxylate groups and ≡FeOH surface sites in the innermost layer, with carboxylates in the outer layer staying in contact with the aqueous medium [33].

The aim of this work is to explore the influence of low-toxicity, low-cost COP dispersion/coating media on the size, size distribution and water dispersibility of iron oxide NPs. Magnetite NPs were prepared by the COP method using food-grade gelatin, agar-agar and a pectin-containing gelling agent. For comparison, magnetite NPs were also prepared in the absence of such additives. The possibility of attaining stable water-based suspensions of these MNPs was assessed, with or without further stabilization with citric acid. CA was used to increase the stability of ferrofluids for MFH measurements. The structure and morphology of the synthesized MNPs were studied by powder X-ray diffraction (XRD), Fourier transform infrared spectroscopy (FTIR) and transmission electron microscopy (TEM). The magnetic properties were studied by SQUID magnetometry and ^57^Fe Mössbauer spectroscopy. To evaluate the potential of the MNPs for MFH, the heating efficiency was determined under an alternating magnetic field, and the results were correlated with the measured magnetic properties.

## 2. Materials and Methods

### 2.1. Sample Preparation

Magnetite NPs were synthesized by the COP method at room temperature, assisted or not by different additives. Except for the additives, all chemicals were analytical grade and used without further purification. Iron (III) chloride hexahydrate ≥99% (FeCl_3_·6H_2_O), iron sulfate heptahydrate ≥99.0% (FeSO_4_·7H_2_O) and citric acid monohydrate ≥99.0% (C_6_H_8_O_7_·H_2_O) were purchased from Sigma-Aldrich Chemicals, Saint Louis, MO, USA, whereas ammonium hydroxide solution 25% (NH_4_OH) was purchased from Honeywell Fluka^TM^, Seelze, Germany. The food-grade additives type B gelatin, pectin-containing gelling agent (Pectigel, Diese) and agar-agar were used as purchased from specialty shops. In the case of Pectigel, additional components are dextrose (33 wt%) and salt (1.6 wt%).

The synthesis of magnetite NPs without the presence of additives closely followed the methodology of Alves et al. [25]. The water used in all preparations was produced by a Milli-Q Academic system from Millipore^®^ (Darmstadt, Germany) (18.2 MΩ cm) and is designated as Milli-Q water.

Stoichiometric amounts of FeSO_4_·7H_2_O and FeCl_3_·6H_2_O were dissolved under a nitrogen atmosphere (to avoid oxidation of the ferrous ions) and magnetically stirred. An excess volume of NH_4_OH was abruptly added to precipitate the NPs, and magnetic stirring was maintained for 60 min under a nitrogen flow. The formed brown-black precipitate was then magnetically separated and washed several times until neutral pH was reached. The resulting product was oven-dried at 60 °C. To infer reproducibility, several samples were produced: two of them, M1 and M2, were chosen to illustrate the results.

Synthesis conditions for the other samples were slightly modified to incorporate the additives. Three samples were synthesized in the presence of a single additive: M–g, M–p and M–a, for m_additive_/m_MNP_ = 0.4. Around 2 g of gelatin (M–g), Pectigel (M–p) or agar-agar (M–a) was previously dissolved in 100 mL of Milli-Q water under magnetic stirring for 60 min, from which a total of 40 mL was added to the reaction vessel. Additives were added in two steps, 20 mL prior to the addition of NH_4_OH and the remainder around 5–10 min after base addition. Two additional samples were prepared with mixed media: pectin–gelatin (M–pg) and pectin–agar-agar (M–pa), both at a 2:1 additive ratio. Table 1 summarizes the sample nomenclature.

The various starting powders were then dispersed in Milli-Q water with the help of ultrasound for 20–30 min and allowed to rest for 5 min. Suspensions that were visibly unstable were decanted and quickly measured, whereas the few with some apparent stability were further centrifuged to remove the larger agglomerates (M–p and M–pa). The recovered supernatants are denoted with an “_MQ” extension.

The basic methodology used to prepare citric acid-based aqueous suspensions with high stability was adapted from those previously described by Campelj et al. and by Kralj et al. [30,32]. In summary, the various MNPs were initially dispersed in Milli-Q water with the help of ultrasound, followed by continuous magnetic stirring. An adequate volume of an aqueous stock solution of citric acid (10 wt%) was then added to each suspension. The pH of the suspensions was initially increased to ca. 5 by dropwise addition of NH_4_OH, followed by 30 min of heating at 60–70 °C. The suspension was cooled down to room temperature, after which the pH was increased to ca. 7, again by dropwise addition of NH_4_OH. All suspensions were then centrifuged for 10 min at 4000 rpm to remove any agglomerated particles. The recovered supernatants are hereafter denoted with a “_CA” extension.

### 2.2. Experimental Methods

To check the crystal structure and phase purity of the obtained products, powder X-ray diffraction (XRD) measurements were performed with a Philips Analytical PW 3050/60 X’Pert PRO (θ/2θ) diffractometer (Almelo, The Netherlands) equipped with an X’Celerator detector and with automatic data acquisition (X’Pert Data Collector software v2.0b (Panalytical, Almelo, The Netherlands). Monochromatized Cu-Kα radiation was used as the incident beam. The diffractograms were acquired in a 2θ range from 10° to 80°, with a 2θ step size of 0.017°. A zero-background holder made of Si crystal was used. The cell parameters were calculated using the Checkcell program (INPG, Grenoble, France) [34].

Fourier transform infrared (FTIR) spectra were recorded on a Thermo Nicolet 6700 FTIR spectrometer (Waltham, MA, USA) for samples dispersed in KBr pellets. The spectra were collected with a resolution of 4 cm^−1^ and 128 scans per sample in the 400–4000 cm^−1^ region.

The morphology of MNPs in the colloidal suspensions was studied by transmission electron microscopy (TEM) using a Tecnai G2 Spirit BioTWIN Transmission Electron Microscope (from FEI, Hillsboro, OR, USA) with digital image acquisition. The particle size distribution (PSD) of the grid-dried suspensions was determined from the TEM images using the program ImageJ [35] and measuring, whenever possible, the diameter of ca. 350 nanoparticles across various regions of each sample. For most _MQ samples, it was not possible to accurately measure individual MNPs due to agglomeration.

The total Fe content in each aqueous suspension was determined by inductive coupled plasma–optical emission spectrometry (ICP-OES) analysis and/or an alternative lab-based method developed for this determination, from here on referred to as LabCal. The latter consisted of selecting adequate volumes of each suspension, which were initially dried and then further calcined at 500 °C, leading to the formation of a stable Fe-oxide phase—hematite (α-Fe_2_O_3_). Once the sample purity and oxide structure were confirmed by means of XRD, the initial Fe content was determined from the obtained oxide mass.

The zeta potentials (ζ) were measured for suitably diluted suspensions at their stabilization pH using a Zetasizer Nano ZS from Malvern Instruments (Malvern, United Kingdom). Samples deemed too unstable were not measured.

Magnetization measurements were performed on a SQUID magnetometer (Quantum Design MPMS, San Diego, CA, USA) after cooling from room temperature in zero magnetic field (zero-field cooling—ZFC) and after cooling under a measurement field of 2 mT (field cooled—FC). Isothermal hysteresis cycles, under applied magnetic fields up to 5.5 T, were obtained at room temperature for the starting powders and at 250 K for the ferrofluids.

^57^Fe Mössbauer spectra were collected at room temperature in transmission mode using a conventional constant-acceleration spectrometer and a 1.85 GBq ^57^Co source in a Rh matrix. The velocity scale was calibrated using an α-Fe foil. The spectra were fitted using the WinNormos Program v3.0 (WISSEL, Ortenberg, Germany), assuming distributions of the magnetic hyperfine field.

The heating efficiency was evaluated by determining the specific loss power (SLP), the power losses under an AC magnetic field normalized by mass unit, for each ferrofluid. In this work, the various suspensions were measured without further preparation, under an AC magnetic field with 276 kHz frequency and 14 kAm^−1^ amplitude, produced with an Easy Heat 0224 device (Ambrell, Almelo, The Netherlands). A volume of 3 mL of each sample was submitted to the oscillating magnetic field for a total of 300 s while recording the temperature of the suspension with an optical fiber temperature sensor (0.1 °C accuracy). To reduce energy exchanges with the surrounding coil and the environment, the induction heating system was isolated and optimized as described elsewhere [22].

## 3. Results

### 3.1. Starting Powders

#### 3.1.1. XRD and FTIR Characterization

**Figure 1:**
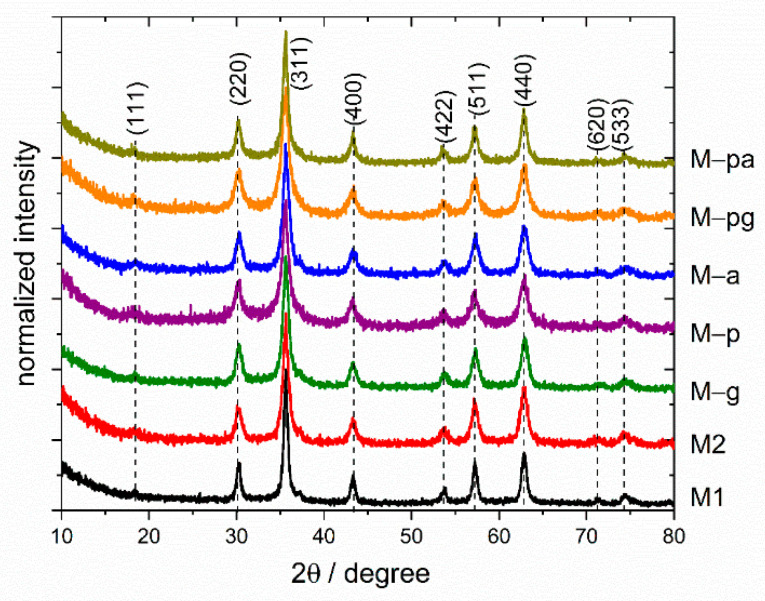
XRD patterns of iron oxide nanoparticles synthesized using a co-precipitation method. The diffractograms were normalized by the maximum of (311) diffraction in all cases, and the base line was displaced between every curve for better view.

Powder X-ray diffractograms of the as-synthesized powders are shown in Figure 1. Within the detection limit, all patterns revealed the presence of a single spinel phase typical of magnetite (S.G. Fd3m, JCPDS No. 89-0691). The determined mean NP size values (<D_XRD_>) and cell parameters are shown in Table 2.

For all samples, the determined cell parameters are between those of bulk magnetite (a = 8.396 Å) and bulk maghemite (a = 8.346 Å). These results indicate sample compositions that are intermediate between both phases and are better described as Fe_3−x_O_4_ [25,36].

The average crystallite size of all starting powders (Table 2) was calculated from the broadening of the main XRD peak, after instrument broadening correction using a silicon sample as a standard crystallite, with the Debye–Scherrer equation and assuming a spherical shape: <D_XRD_> = 0.89 λ/(βcos θ), where λ is the wavelength of Cu-Kα radiation and β is the full width at half maximum of the peak in radians.

**Figure 2:**
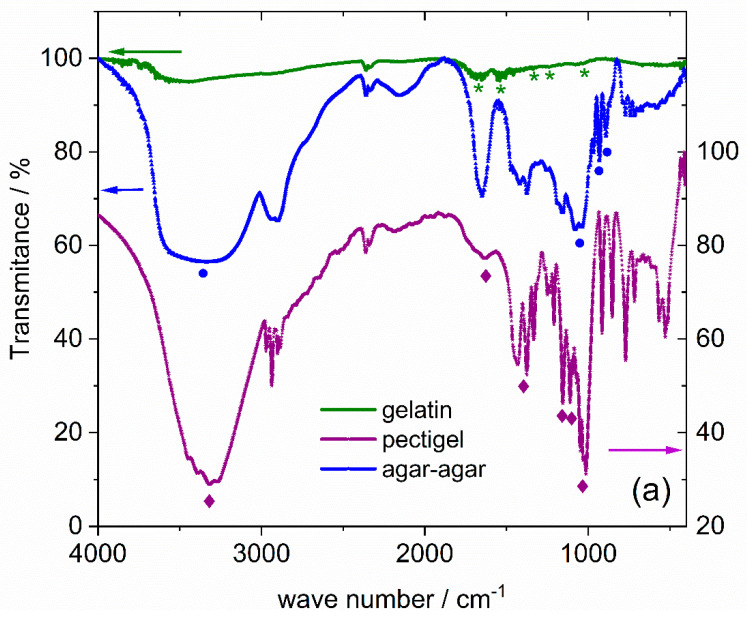

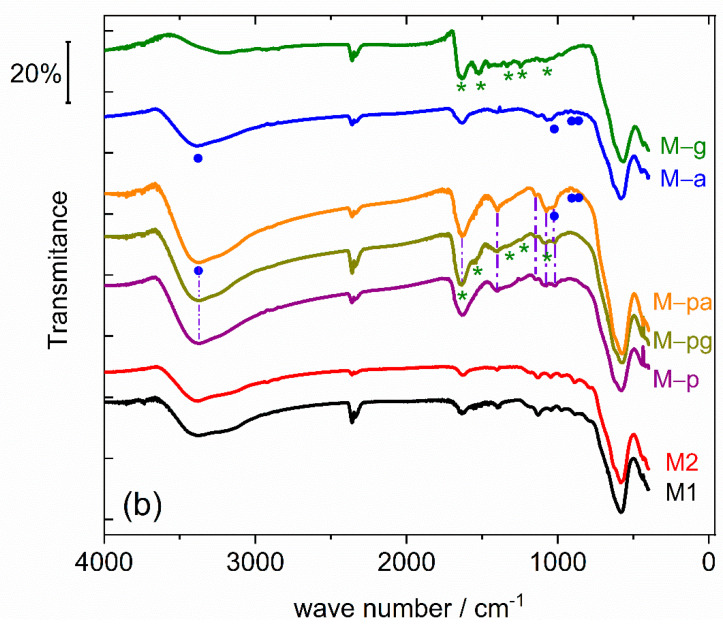
(**a**) FTIR spectra of gelatin, agar-agar and Pectigel (pectin-containing food-grade additive) and (**b**) of the various synthesized powders. Some of the spectra are displaced vertically for better visualization, and the peaks considered associated with the different additives are marked by green stars (gelatin), blue circles (agar-agar) and violet diamonds/dash-dot lines in the case of Pectigel/pectin.

Figure 2 displays the FTIR spectra, the transmittance as a function of the wave-vector, of the as-purchased additives and synthetized powders, respectively. All spectra show a minimum around 2360 cm^−1^ due to background CO_2_. All synthesized MNPs exhibit absorptions around 580 and 440 cm^−1^, which are attributed to Fe-O bonds for iron in tetrahedral and octahedral coordination, respectively, and a small shoulder around 668 cm^−1^. This agrees with reported results for γ-Fe_2_O_3_ NPs that indicate three main bands at 637, 557 and 442 cm^−1^ [37].

In the bare MNP results, the absorption band around 3400 cm^−1^ can be assigned to the stretching vibration of O-H surface groups, and the absorption around 1630 cm^−1^ can be attributed to the bending mode of adsorbed water [38]. The other very weak absorptions at 1130, 1044 and 974 cm^−1^ are attributed to adsorbed residuals from the synthesis, namely, adsorbed sulfate ions [39].

Gelatin has weaker absorptions than the two other additives. Nevertheless, the FTIR results for the gelatin and the gelatin-prepared sample, M–g, share several absorptions between 1000 cm^−1^ and 1700 cm^−1^. These are marked with a green star in the referred spectra and also in the gelatin spectra and were identified by the reported absorptions at 1663 cm^−1^ for C=O stretching (amide I)/carboxylate, 1532 cm^−1^ for NH bending coupled with C-N stretching (amide II), 1449 cm^−1^ for CH_2_ bending of amides, 1252 cm^−1^ for NH bending and 1075 cm^−1^ for C-O stretching [38,40]. Additionally, the broad absorption around 3200 cm^−1^ is attributed to N–H stretching and C-H stretching absorptions centered, respectively, at 3447 cm^−1^ and 3068 cm^−1^. Thus, the FTIR results corroborate the existence of gelatin at the surface of the M–g sample.

Agar-agar is a hydrophilic polysaccharide composed of agarose and agaropectin. The former consists of an agarobiose repeating unit, α-1,3-linked D-galactose and β-1,4-linked 3,6-anhydro-L-galactose, whereas the latter is the sulfated form of agarobiose [41]. The non-gelling fraction, agaropectin, is often removed in commercial agar-agar to improve its properties, accounting for roughly one-third of the natural agar-agar [42,43]. Comparing the FTIR spectrum of the agar-agar-prepared sample, M–a, with that of the as-purchased food-grade agar, both share a broad absorption band centered at 3390 cm^−1^ associated with O-H stretching vibration, a strong absorption close to 1650 cm^−1^ corresponding to amide I vibrations [44] and broad absorption close to 1060 cm^−1^ attributed to C-O and C-C stretching and C-O-H bending modes [45,46]. The absorption at 931 cm^−1^ may be attributed to the C-O stretching of 3,6-anhydro-galactose, and the one at 891 cm^−1^ can be ascribed to the stretching of C-H residual carbon of β-galactose [47]. The three last absorptions do not superimpose those of the bare NP spectra and are identified in M–a and agar-agar spectra by blue circles. Thus, the FTIR results confirm the presence of an agar–MNP composite in the M–a sample.

The gelling agent Pectigel contains pectin and dextrose. As a commercial gelling agent, it is expected to have pectin mixed with a significant amount of its weight in sugar, typically dextrose, to facilitate dispersion in solution [48]. Pectin is a linear polysaccharide and consists mainly of 1,4-α-D-galacturonic acid molecules with carboxyl/hydroxyl groups distributed along the backbone. Some of the carboxyl groups occur naturally as methyl esters, whereas exposure to ammonia may convert some of its methyl ester groups into amide groups (amidated pectin) [49]. On the other hand, dextrose is a simple hexose monosaccharide. The FTIR spectrum of the as-purchased additive Pectigel has an intense and broad absorption band centered around 3338 cm^−1^ associated with the O-H stretching of hydroxyl groups, a significant broad absorption at 1636 cm^−1^ attributed to the C=O stretching of carboxylate groups [50] and absorption around 1400 cm^−1^ attributed to the symmetric stretching of the carboxyl group [45] or stretching and bending modes of C-H and C-O, with possible contributions from O-H bending. The sharp and intense absorption at 1027 cm^−1^ in the FTIR spectrum of the additive Pectigel (Figure 2a) is ascribed to the glycosidic bond in pectin [50,51,52,53,54] and can also be detected in M–p. For MNP synthetized with the assistance of Pectigel (single or mixed with another additive), the FTIR spectrum exhibits, in addition to strong Fe–O-related bands, a series of absorptions that can be related to this additive, albeit exhibiting some differences in relation to pure Pectigel spectra, namely, the significant increase in the relative intensity of the 1631 cm^−1^ absorption, combined with the almost total disappearance of the ester carbonyl vibration (found at 1723 cm^−1^ in Pectigel), which seems to point to a significant degree of de-esterification of the pectin polymer chain. This phenomenon has been previously reported and interpreted as a sign of successful pectin amidation [55]. A significant decrease in relative intensity around 1020 cm^−1^ is also seen and may indicate a degree of de-polymerization of the pectin chain, which is expected to be lower for NH_4_OH-treated pectin when compared to NaOH [21]. The new absorptions, or the ones that increase in the spectra of samples synthesized with Pectigel in comparison with M1 and M2 spectra, are marked with vertical dash-dot lines, indicating the presence of pectin in M–p, M–pg and M–pa and the formation of the corresponding MNP/biopolymer composites. The corresponding absorptions in Pectigel spectra are marked with violet diamonds. In M–pa, due to the chemical similarity of agar-agar and pectin, assessing the presence of both additives is not straightforward. However, the increased absorption in the band between 1125–970 cm^−1^ with the presence of weak absorptions at 930 and 891 cm^−1^, attributed to the presence of 3,6-anhydro-galactose residues and C-H bending at the anomeric carbon in β-galactopyranosyl residues, respectively, points to the presence of a pectin/agar/MNP composite. Table 3 summarizes the most important bands and corresponding vibration modes for Pectigel-assisted synthesized NPs.

#### 3.1.2. Mössbauer Spectroscopy Study

**Figure 3:**
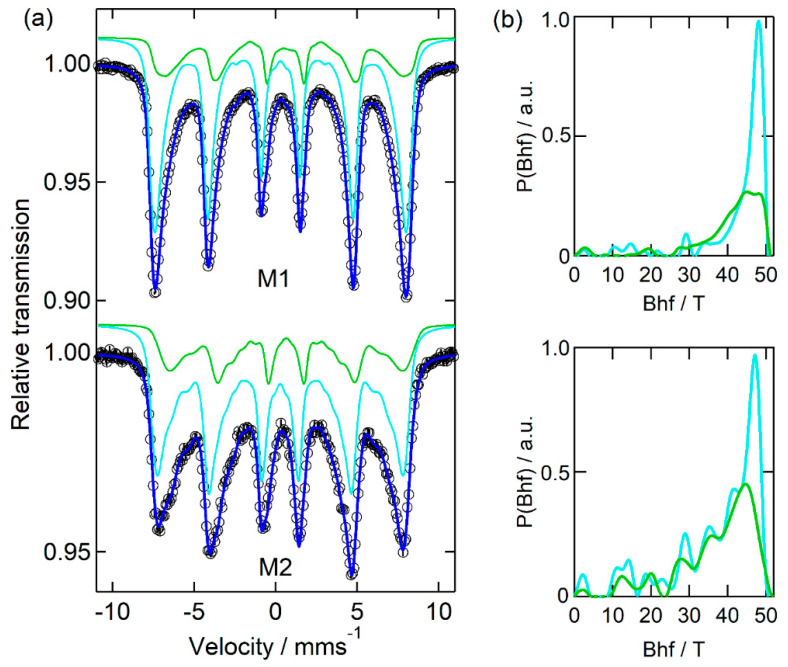
(**a**) Mossbauer experimental spectra (open circles) and fitted curves (blue lines) of the as-prepared MNP M1 and M2 (without additives). Cyan and green lines correspond to the contributions of the magnetic hyperfine interactions in site 1 and site 2, respectively, associated with different oxidation states of iron. The corresponding distributions of the magnetic hyperfine fields are plotted in (**b**), using the same colours.

**Figure 4:**
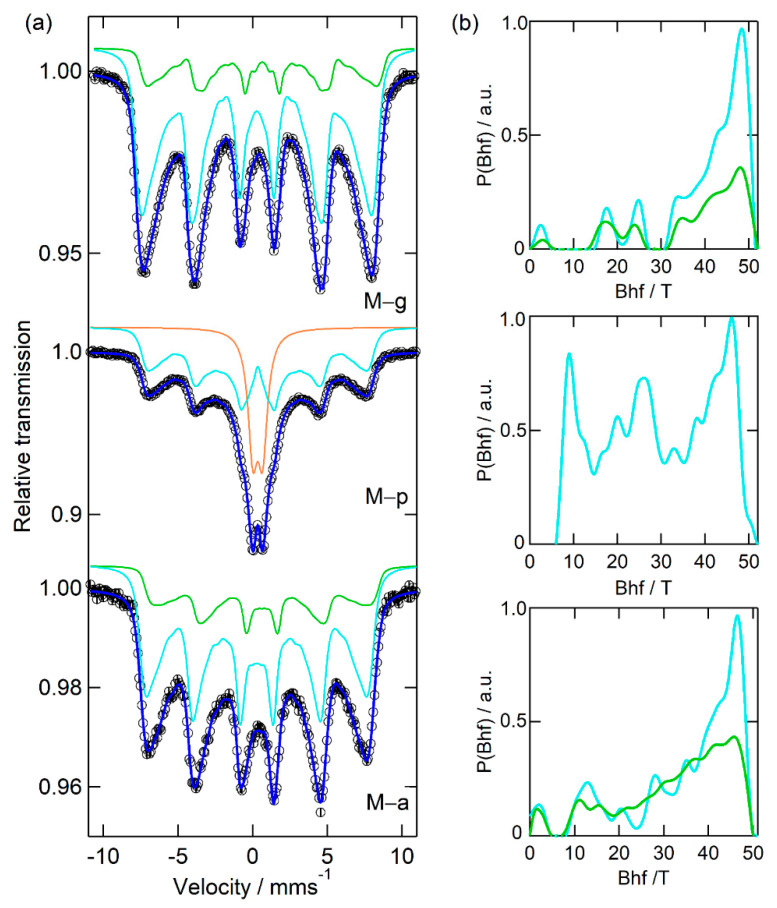
(**a**) Mossbauer experimental spectra (open circles) and fitted curves (blue lines) for samples synthetized with the aid of gelatin (M–g), Pectigel (M–p) and agar-agar (M–a) additives. Cyan and green lines correspond to the contributions of the magnetic hyperfine interactions in sites 1 and 2, respectively, associated with different oxidation states of iron. The corresponding distributions of the magnetic hyperfine fields are plotted in (**b**), using the same colours. For M–p, the component fitted with a quadrupole doublet is drawn with an orange line and is associated with a superparamagnetic behavior of the smaller NP.

**Figure 5:**
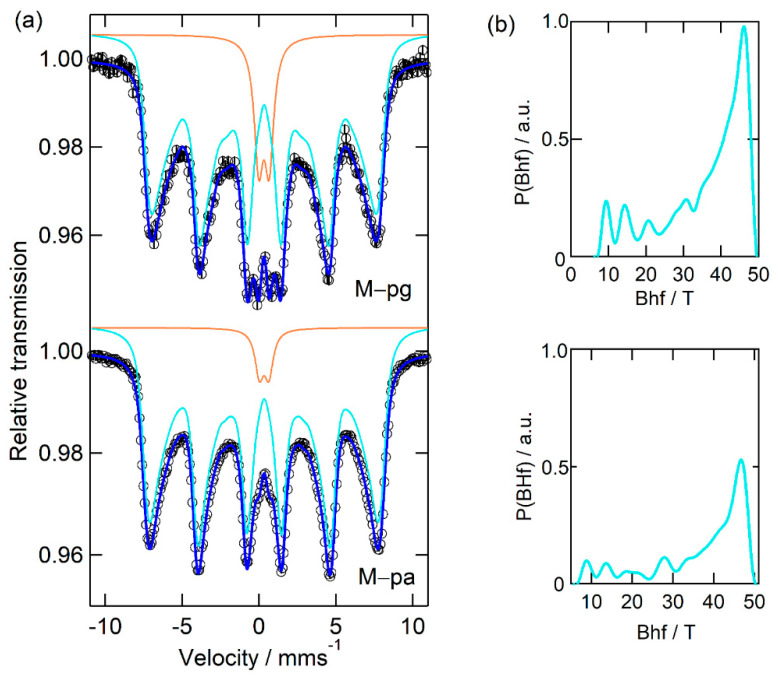
(**a**) Mossbauer experimental spectra (open circles) and fitted curves (blue lines) for samples synthetized with the assistance of mixed media at 2:1 ratio: pectin–gelatin (M–pg) and pectin–agar-agar (M–pa). The cyan lines correspond to the contribution of magnetic hyperfine interactions and are associated to the magnetic hyperfine field distributions shown in (**b**). The orange lines represent the components fitted with quadrupole doublets and are related to a superparamagnetic behavior of the smaller NP.

Figure 3, Figure 4 and Figure 5 display the results obtained from measuring all of the starting powders by ^57^Fe Mössbauer spectroscopy, whereas Table 4, Table 5 and Table 6 present the corresponding hyperfine fitting parameters for the various samples.

Figure 3 and Table 4 show that both spectra of the MNPs synthesized without the presence of additives are well resolved, considering the two iron sites and distributions of the hyperfine magnetic field (B_hf_). The two iron sites exhibit different isomer shift (IS) values. The one with a higher mean field (site 1—cyan B_hf_ distribution) presents IS values of around 0.28 mm s^−1^ and is assigned to Fe^3+^; the one with lower <B_hf_> (site 2—green B_hf_ distribution) has IS values of around 0.60 mm s^−1^ and is a typical signature of Fe^2.5+^ in magnetite (mixture of Fe^2+^ and Fe^3+^) [57]. However, none of the samples contain sextets with the relative amount of Fe^3+^ and Fe^2.5+^ corresponding to the bulk magnetite characteristic occupation of tetrahedral and octahedral iron sites [Fe^3+^]_tet_[Fe^2+^,Fe^3+^]_oct_O_4_, indicating a certain degree of oxidation and confirming that the samples are better described as Fe_3−x_O_4_, in accordance with the XRD results. The broad hyperfine field distributions obtained for both iron sites in M1 and M2 samples are attributed to the NP size distributions, since iron nuclei in NPs with different sizes will experience diverse mean hyperfine fields and different magnetic relaxation times. In addition, for small NPs, iron oxidation will contribute to broadening the site 1 distribution (associated with Fe^3+^) and to decreasing the contribution of the site 2 component. It should be noted, however, that neither M1 nor M2 spectra can be properly fitted without the higher IS component. A range of very low magnetic fields was also considered in the corresponding fitted distributions, as seen in the relative distribution plots, P(B_hf_), for both sites and samples. Although corresponding to a very small contribution when compared with the iron nuclei fraction experiencing higher fields, it improves the fitting quality in the central part of the spectra. A possible explanation is the presence of iron at the nanoparticle surface, associated with random defects. The same good fitting result was not obtained if, instead, a quadrupole doublet was considered. Moreover, considering an equivalent synthesis methodology for samples M1 and M2, the marked differences between the two spectra (much lower mean fields, broader peak width and higher quantity of smaller NPs in M2 in relation to M1) confirm the poor reproducibility of the unassisted COP method.

The Mössbauer results for samples synthetized in the presence of a single additive show a marked variation with the additive itself, with the M–g and M–a spectra sharing some similarities. The results are illustrated in Figure 4 and further detailed in Table 5. M–g and M–a spectra are well reproduced using only two magnetic sextets, with the sub-spectra of M–g having the strongest mean magnetic fields of all MNPs prepared with additive assistance.

With Pectigel, the as-prepared NP (M–p) spectrum displays only one sextet (site 1—cyan B_hf_ distribution), associated with the weakest mean magnetic field, and the typical signature of Fe^2.5+^ is not present. In addition, the spectrum includes a quadrupole doublet (site 3, ca. 30% intensity) that, combined with the magnetization results (see below), is associated with very small particles displaying superparamagnetic behavior within the time scale of a Mössbauer measurement.

From Figure 5 and Table 6, another set of interesting observations can be made, now for the synthesis in mixed media.

Like in M–p, both samples M–pg and M–pa display one sextet of Fe^3+^ (site 1—cyan B_hf_ distribution) and a quadrupole doublet (site 3), although in a smaller fraction than in the M–p sample, consistent with a reduction in the influence of Pectigel due to the presence of the other additive and evidencing the influence of both additives.

Thus, the Mössbauer results indicate that Pectigel-containing media lead to the appearance of very small NPs and the disappearance of the Fe^2.5+^ signature seen for all other samples.

#### 3.1.3. SQUID Magnetometry

**Figure 6:**
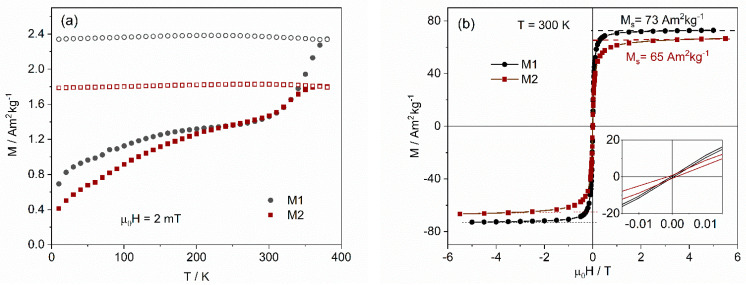

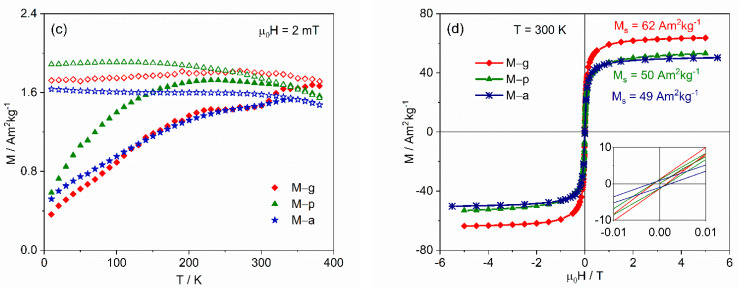
Magnetization results for the MNPs synthesized without additives (**a**,**b**) or with a single additive (**c**,**d**). **Left**—temperature dependence after zero-field cooling (ZFC) (full symbols) and after cooling under a measuring field of 2 mT (FC) (open symbols); **right**—isothermal hysteresis curves at 300 K showing, in the inset, the zoomed low-field region, confirming negligible coercivity.

The magnetization evolution with temperature (ZFC/FC) and room-temperature hysteresis curves for the MNPs prepared without additives or with a single additive are presented in Figure 6. The shape of the curves is characteristic of small single-domain NPs, displaying a broad peak in the range of 150 K to 250 K and, in some cases, a second peak at higher temperature in the ZFC curve. In a collection of monodisperse single-domain NPs, the ZFC is expected to exhibit a peak at a temperature, T_B_, that separates the superparamagnetic state (above T_B_) from the blocked regime (below T_B_). However, in distributed MNPs, the peak occurs at a temperature T_max_ that is slightly higher than the average blocking temperature, depending on the size distribution and shape of the particles [58,59]. Above T_max_, the temperature at which the ZFC and FC curves merge corresponds to the irreversibility temperature, T_irr_, and is identified by the blocking temperature of the biggest particles in the sample. The relative difference between T_max_ and T_irr_ gives a measure of the relative width of the NP volume distribution. The coexistence of two maxima in the ZFC curve is usually observed for NP distributions with an average diameter between 11 nm and 17 nm [36] and is associated with the coexistence of two magnetic iron oxide phases, maghemite for the smaller NPs and magnetite for the larger ones. The volume magnetic anisotropy constant of magnetite is three times larger than that of maghemite, with this difference separating the corresponding blocking temperatures by a finite temperature interval.

Considering that more maghemite is associated with smaller NP with a more important peak at lower temperature, the results indicate that sample M–p presents the largest degree of oxidation, while M–a and M–g display similar magnetic behavior, in good agreement with the Mössbauer results.

From the hysteresis at 300 K, the spontaneous magnetization values, Ms, were obtained, decreasing from 69(4) Am^2^kg^−1^ for the NPs synthesized without any additive to 49(1) Am^2^kg^−1^ for the one synthesized with agar. These values of M_s_ are lower than the bulk magnetite and maghemite values, 92 and 76 Am^2^kg^−1^, respectively [60]. For the uncoated MNPs, M1 and M2, the smaller M_s_ relative to bulk material is explained by the existence of a magnetic dead-layer at the surface due to misalignment of the atomic spins, which is expected to be similar for closely matched NP sizes. The calculation of the thickness, t, of this dead-layer, assuming a spherical maghemite core with radius R, gives $t=\frac{\langle D_{\mathrm{XRD}}\rangle }{2}\left[1-{\left(\frac{M_{s}}{M_{s\;\mathrm{bulk}}}\right)}^{1/3}\right]$ and a value of 0.6 nm for M2. Since the average iron oxide crystallite sizes of the coated NPs, determined by XRD, are similar to those of M2, the decrease in M_s_ for the NPs prepared with additives is an indication that the mass of the coating increases in the order: gelatin < Pectigel< agar. Assuming that the magnetization of the magnetic core of the NPs has a value between that of M2 and that of bulk maghemite, the mass fraction of coating can be calculated by $\frac{m_{\mathrm{coating}}}{m_{\mathrm{NP}}}=1-\frac{M_{s}}{M_{s}\left(\mathrm{core}\right)}$ and would be 11(6)%, 29(6)% and 30(5)%, respectively, for gelatin, Pectigel and agar. The uncertainty is due to the uncertainty of M_s_(core).

The hysteresis curves exhibit coercive fields below 1.2 mT, a value that corresponds to the remnant field of the magnetometer superconductor coil after reaching 5.5 T, indicating that the coercivity is negligible and the NPs can be considered superparamagnetic particles at 300 K.

**Figure 7:**
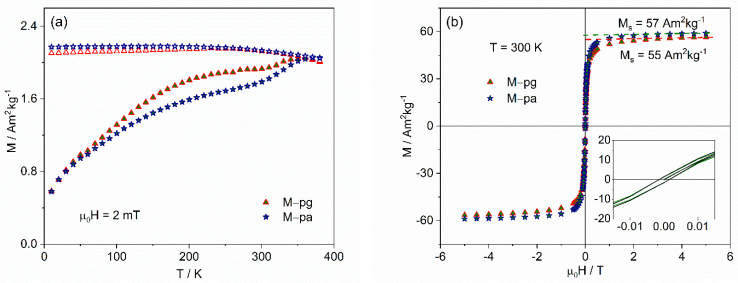
Magnetization results for the MNP synthesized with two additives: (**a**) temperature dependence after zero-field cooling (ZFC) (full symbols) and after cooling under the measuring field of 2 mT (FC) (open symbols); (**b**) isothermal hysteresis curves at 300 K showing, in the inset, the zoomed low-field region, confirming negligible coercivity.

The results for samples synthesized in mixed media, shown in Figure 7, are similar, with spontaneous magnetization values of 55 and 57 Am^2^kg^−1^. Estimating the mass percentage associated with the coating, as before, gives 21(6)% and 19(7)%, respectively.

### 3.2. Characterization of Water-Based Suspensions and Ferrofluids

#### 3.2.1. Zeta Potentials and Fe Loading

Table 7 shows the ζ potentials of the _MQ aqueous suspensions and _CA ferrofluids at their stabilization pH, the mass ratio between added citric acid and suspended MNPs (m_CA_/m_MNP_) and the total Fe concentration obtained by ICP-OES and LabCal methods.

The two methods used to evaluate the iron concentration, C_Fe [ICP]_ and C_Fe [LabCal]_, are in excellent agreement, validating the LabCal method as a valuable method for iron concentration determination in MNPs. The results show that, for similar protocols, the total Fe concentration is lowest for the water-based suspensions (_MQ), followed by the _CA ferrofluids obtained with uncoated NPs (M1_CA and M2_CA). The remainder of the samples reached total iron concentrations between 4.3 and 7.7 mg/mL (LabCal).

The zeta potential essentially predicts the stability of a colloidal suspension, with absolute values greater than 30 mV indicating a high degree of stability [61]. From the original powders, only M–p and M–pa produce sufficiently stable suspensions in water (M–p_MQ and M–pa_MQ) to undergo a zeta potential measurement, giving values of −36 mV and −37.8 mV, respectively. All the other samples suspended in water were deposited after a few minutes. The group of _CA ferrofluids should be considered separately due to the presence of biopolymer coatings and the different stabilization media between NPs [62]. The comparison of zeta potentials within this group clearly indicates a dependence on the coating. For coated NPs, better stability was obtained for M–p_CA and M–a_CA samples. The stability of the ferrofluids was also assessed by evaluating if noticeable deposition could be observed on a time scale of several days and months. No appreciable deposition was seen even after ca. 12 months of storage, allowing confirmation of the good suspension stability of all samples but M–g.

#### 3.2.2. Particle Size and Size Distribution

**Figure 8:**
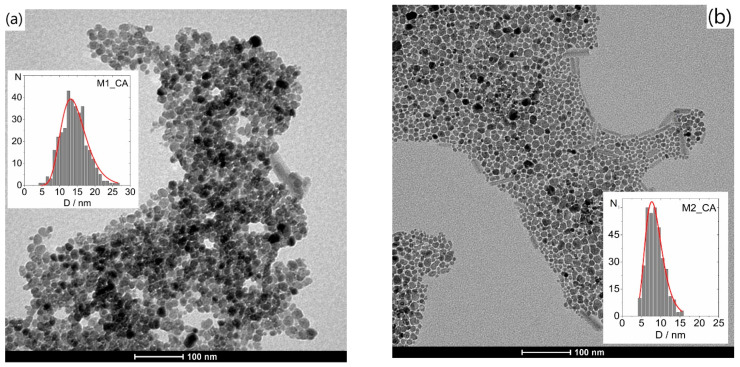
Typical TEM images of the grid-dried citric acid-based ferrofluids and corresponding size distributions for samples (**a**) M1_CA and (**b**) M2_CA.

**Figure 9:**
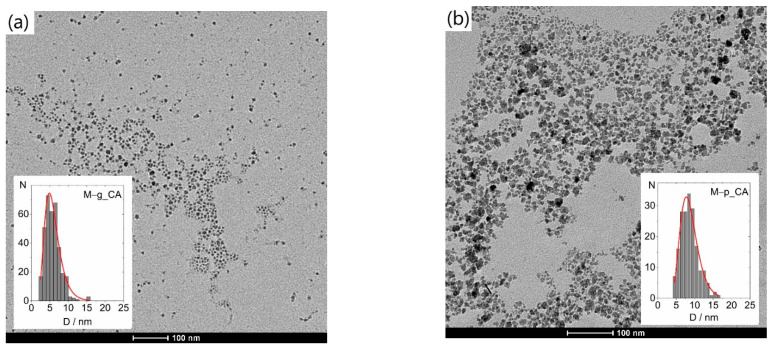

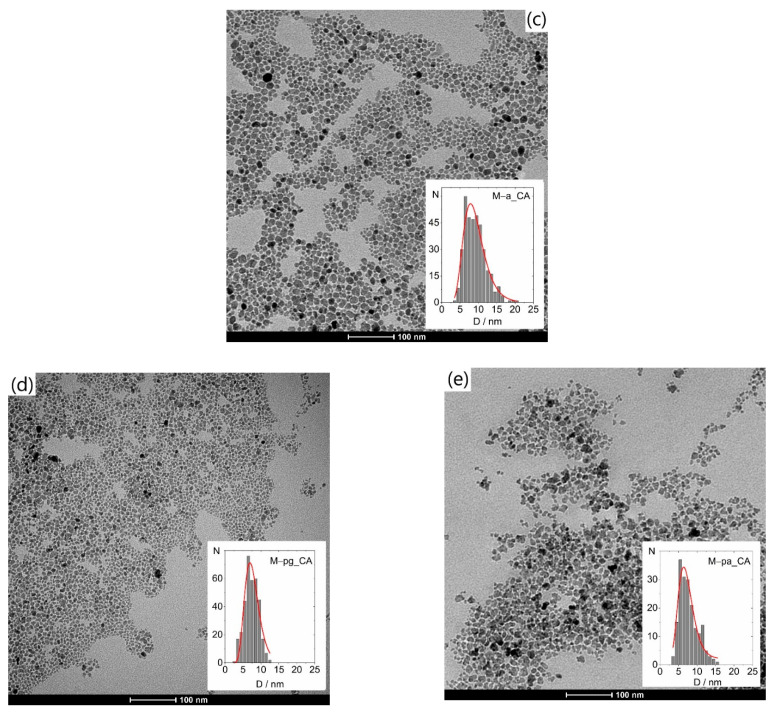
Typical TEM images of the grid-dried citric acid-based ferrofluids with the corresponding size distributions for coated NPs in samples (**a**) M–g_CA, (**b**) M–p_CA, (**c**) M–a_CA, (**d**) M–pg_CA and (**e**) M–pa_CA.

**Figure 10:**
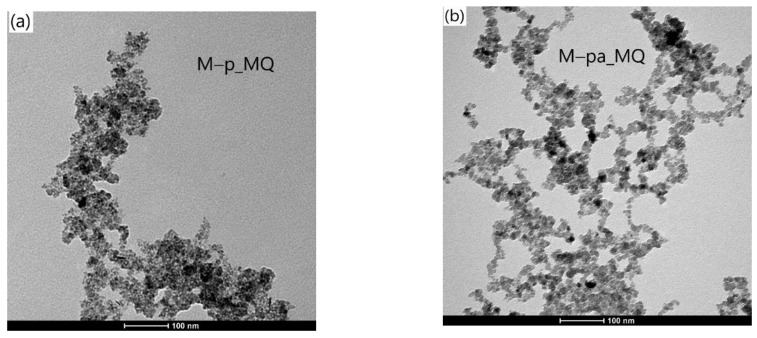
Typical TEM images of the grid-dried water dispersions for (**a**) M–p_MQ and (**b**) M–pa_MQ.

Figure 8, Figure 9 and Figure 10 show representative TEM images and size distributions of the grid-dried CA-based and water ferrofluids (_MQ). In the latter, agglomeration after drying inhibited particle counting, disabling further statistical analysis. Table 8 summarizes the determined mean particle size (<D_TEM_>) and standard deviations from fitting Log-Normal distributions to histograms of ~350 NPs from different areas of various TEM images.

The <D_TEM_> values obtained for the different samples follow the same relative trend as <D_XRD_>, with the former being generally smaller.

The uncoated M1_CA and M2_CA samples with nearly spherical NPs (Figure 8) show significant differences in both size and size distribution, despite having nominally equivalent synthesis methodologies, with <D_TEM_> of 15 ± 4 nm and 9 ± 2 nm, respectively.

The M–g_CA image in Figure 9 reveals very small and irregularly shaped NPs, with <D_TEM_> of 6 nm compared to <D_XRD_> of 11 nm, a size reduction of ca. 50%. This significant reduction is addressed later.

The differences between MNPs obtained with Pectigel loaded in the synthesis medium and those obtained with agar-agar lie in the shape rather than the size or size distribution. The TEM images show highly irregular shapes for M–p and near-spherical shapes for M–a. The irregular shape of the M–p nanoparticles, not observed for other additives, can be explained by different functionalization reaction rates associated with different viscosities of the additive solutions (not measured) and by the pectin structure, which can present differently sized cavities. The latter is justified by the intramolecular interactions in pectin between COOH and OH groups. Only M–p and M–pa enabled stable suspensions without the addition of citric acid.

#### 3.2.3. SQUID Magnetometry

**Figure 11:**
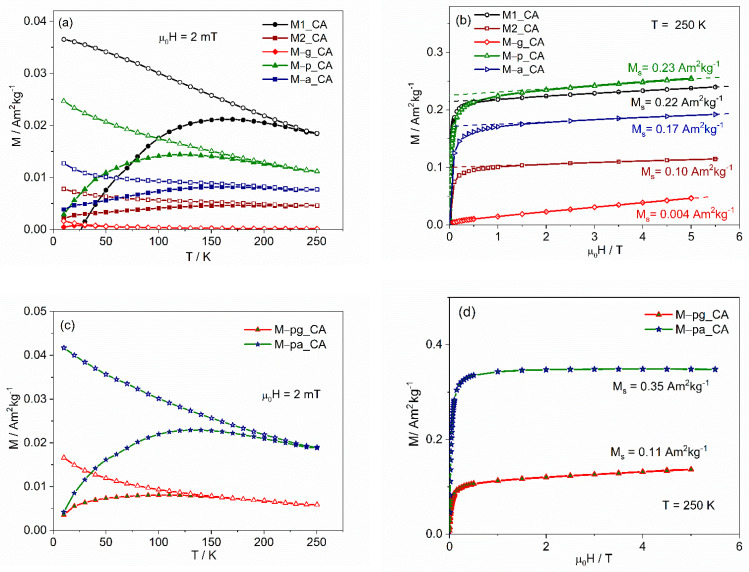
Magnetization results for the ferrofluids: (**a**,**c**) temperature dependence after zero-field cooling (ZFC) (full symbols) and after cooling under the measuring field of 2 mT (FC) (open symbols); (**b**,**d**) isothermal magnetization curves at 250 K.

The ZFC/FC magnetization curves for the citric acid–stabilized ferrofluids, corrected by the diamagnetic contribution of water, show single-domain magnetic behavior (Figure 11a,c) with a blocking temperature smaller than those of the original powders, characteristic of smaller sized MNPs and in good agreement with the trend seen in Table 8 for <D>. The low-temperature marked increase in the FC curve with decreasing temperature indicates a significant paramagnetic contribution, as is also seen in the magnetic field dependence (Figure 11b,d) as an asymptotic linear dependence at high fields. This paramagnetic behavior can be associated with very small NPs with diameters below 3 nm [63]. Defining magnetic NPs as those that contribute to the ferrimagnetic hysteresis curve at 250 K, their concentration in the ferrofluid can be determined from the magnetization curves (Figure 11b,d). From the spontaneous magnetization, obtained by extrapolating the linear behavior at high fields to zero magnetic field, and considering a magnetization value of 50 Am^2^kg^−1^ (characterizing 7–8 nm bare MNPs [36]), the mass concentration of ferrimagnetic magnetite/maghemite NP was calculated. These values, C_iron oxide_, are presented in Table 9. The concentration of iron in the MNPs, C_Fe(MNP)_, also shown, was calculated using the iron-to-iron-oxide mass ratio, which is similar in magnetite and maghemite. A straightforward conclusion from these results is that not all of the iron in the ferrofluids is in the MNPs. This means that in the preparation of citric acid–stabilized ferrofluids, both for uncoated and for coated NPs, a fraction of the original NPs was dissolved and/or the fraction left of the NPs remaining after centrifugation is too small to behave ferrimagnetically.

The role of citric acid in NP dissolution is clearly demonstrated by comparing the magnetic fractions for aq. citric acid–stabilized suspensions M–p_CA (44%) or M–pa_CA (64%) with those for the corresponding water suspensions M–p_MQ (100%) and M–pa_MQ (98%). A significant reduction in concentration is obtained in the former, while no loss is accounted for in the latter.

It is known that low pH values favor the dissolution of MNPs due to the weakening of the Fe–O bond by a protonation mechanism [53]. In addition, Szekeres et al. [64] showed that the addition of CA at 1.6 mmol COOH/g MNPs reduced the particle diameter from 8 nm to ca. 7.2 nm, with subsequent formation of iron–citrate complexes or iron hydrolysis products, indicating that citric acid enhances the iron dissolution. In our method, the much higher ratios used and the intermediate adsorption step at 60–70 °C can enhance citric acid NP corrosion.

No significant differences for the CA-ferrofluids were determined between the as-prepared state and their state 6 to 12 months after their preparation, indicating that dissolution occurred during the initial steps of the CA stabilization process, where pH ranged between 2 and 5.

Ordering the coatings in terms of remaining magnetic iron oxide fraction (F_mag_) for the coated NPs leads to F_mag_(gelatin) << F_mag_(Pectigel) < F_mag_(agar), which corresponds to the ordering for the coating mass predicted for the original powders. Thus, it could be tempting to explain the results as dependent on the degree of protection given by the biopolymer layer against citric acid–induced dissolution/corrosion (the thicker the coating, the larger the protection). However, ferrofluids with uncoated NPs were affected the least, although they were also affected, so the enhanced effect in the presence of additive coatings, which is more extreme for the gelatin additive, requires an additional mechanism.

Gelatin, pectin and agar-agar are hydrophilic but can change their hydrolytic stability by crosslinking. Citric acid is being increasingly chosen as a crosslinking agent for various polysaccharides and proteins [65,66]. The citric acid stabilization protocol used in our ferrofluid preparation probably induced the crosslinking of the biopolymer surface coatings, changing their hydrophilicity in an irreversible way. This effect was more important in M–g_CA, a result that can be explained by the covalent crosslinking and insolubility induced by gelatin’s high degrees of dehydration (less than 2% moisture level) [67] and heat-induced hydrophobicity [68]. The resulting poorer water dispersibility implies a much larger removal of MNPs during the final centrifugation step. Thus, the final magnetic fraction in the CA ferrofluids is the result of the interplay between CA-induced MNP dissolution during the low pH stage, CA-induced crosslinking and centrifugation during the ferrofluid preparation.

For the ferrofluids prepared without citric acid, a degree of thermal crosslinking (during oven drying at 60 °C) and/or thermal degradation may be responsible for the poor dispersibility of gelatin- and agar-agar-coated NPs in water. That was not the case for the ones prepared using Pectigel as the additive, samples M–p_MQ and M–pa_MQ, which exhibit excellent stability in water.

#### 3.2.4. Magnetic Hyperthermia

**Figure 12:**
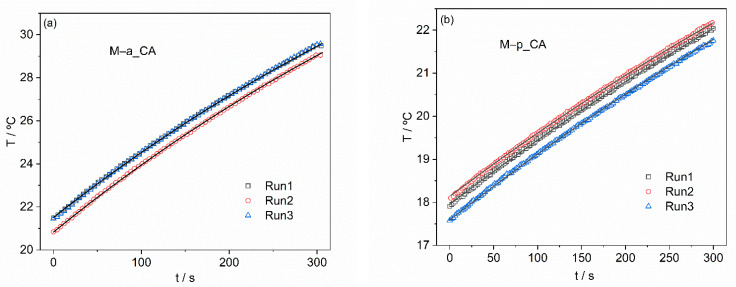
Temperature evolution during applied ac magnetic field for samples: (**a**) M–a_CA and (**b**) M–p_CA, showing good reproducibility of the results and the good quality of the fit.

The SLP was determined for all ferrofluids to evaluate their heating efficiency, as explained before. At least three heating cycles were performed for each sample to confirm the reproducibility of the results, starting at the same temperature. In general, heating periods of 300 s were used (illustrated in Figure 12 for M–a_CA and M–p_CA), except in the case where deposition was visible during this period, the case of naked NPs, samples M1_CA and M2_CA, a situation where the heating period was reduced to 100 s. The reproducible temperature versus time curves were used to determine the power dissipated by the MNPs by fitting the results with an expression that assumes constant power dissipation when the magnetic field is applied and a small energy loss to the environment, which is linear with the temperature difference between the sample and environment. Our method has been validated in collaboration with several laboratories under RADIOMAG EU COST action TD 1402 [69]. The SLP results obtained used the iron oxide mass calculated from the magnetization results (Table 9) and are presented in Table 10. To compare results obtained with different ac magnetic fields, meaning different frequencies and/or amplitudes, the intrinsic loss power (ILP), defined as the SLP divided by the frequency and the square of the amplitude of the magnetic field, is also presented in Table 10. With pectin-containing coatings, very good ILP values were obtained, between 2.8 and 4.7 nWg^−1^.

## 4. Conclusions

The results indicate that the use of current additives can be a way to control the size reproducibility of MNPs produced via a COP route when compared with COP without any additives.

An easy, low-cost method to determine the iron content of the iron oxide nanoparticles is proposed, using the calcination of a known sample mass to totally convert it to hematite. This method was used and validated by comparing the data with ICP results from the same samples.

The presence of additives in the coated MNPs was confirmed by FTIR. When comparing the different MNPs synthesized with only one additive, the pectin coating is the one that provides the best stability for the NP suspensions in water. Additionally, the combination of pectin with agar-agar results in a stable water dispersion of the NPs. The use of these coatings configures an easy way to obtain a controlled level of hydrophobicity without toxic or expensive organic solvents. In this work, sample stability was retained for several months without an additional crosslinking step.

The stabilization of water suspensions using citric acid in combination with centrifugation resulted in a significant decrease in the size of the MNPs, the effect being more important in the case of gelatin-synthesized samples. The total iron content of the _CA samples is very different from the iron content of the MNPs, determined using the magnetic characterization, contrary to the case of _MQ samples. Accordingly, the _CA samples contain very small NPs, without significant contribution to the magnetization or to the heat dissipation under an ac magnetic field. These results are a consequence of the ferrofluid preparation method and are explained by the interplay between CA-induced MNP dissolution, biopolymer crosslinking and centrifugation. After preparation, the stability and physical properties of the suspensions remain unchanged.

For stable suspensions prepared with citric acid, better heating efficiency was attained for pectin-containing coatings, giving ILP values between 2.80 and 4.74 ${\mathrm{nWg}}_{\mathrm{MNP}}^{-1}$. In the case of the stable water-based suspensions, ILP values of 1.54 and 3.60 ${\mathrm{nWg}}_{\mathrm{MNP}}^{-1}$ were obtained for pectin and pectin–agar coatings, respectively. The higher ILP values obtained are in accordance with the values presented in the literature for commercial nanoparticles of similar sizes that, in several cases, do not present better values [70,71].

## Figures and Tables

**Table 1:** Sample nomenclature of the starting powders.

Medium Type	Sample
None	M1
None	M2
Gelatin	M–g
Pectigel	M–p
Agar	M–a
Pectigel–Gelatin	M–pg
Pectigel–Agar	M–pa

**Table 2:** Mean particle sizes (<D_XRD_>) and cell parameters (a) obtained from XRD.

Sample	<D_XRD_>/nm	a/Å
M1	16	8.352(4)
M2	12	8.372(7)
M–g	11	8.356(2)
M–p	10	8.366(5)
M–a	11	8.351(3)
M–pg	9	8.355(5)
M–pa	12	8.349(2)

**Table 3:** Main bands and corresponding vibration modes in the FTIR of magnetic nanoparticles synthesized in the presence of Pectigel food additive, either alone or in mixed media with food-grade gelatin or agar. vs = very strong; s = strong; m = medium intensity; w = weak; sh = shoulder.

Wavenumber/cm^−1^			Vibration Mode
M–p	M–pg	M–pa	
3374 (vs)	3330 (vs)	3379 (vs)	O-H stretching
1631 (s)	1636 (s)	1631 (s)	C=O asym. stretching (carboxylate)/amide I
-	1546 (m)	-	NH bending/C-N stretch (amide)
1400 (m)	1401(m)	1398(m)	C=O sym. stretching carboxylate; C-H bending; O-H bending
1086 (m)	1080 (m)	1063 (m)	C-O stretching
-	-	1045 (m)	C-O stretching, C-C/C-O-H bending [45]
1021 (m)	1021(m)	1021 (m)	Glycosidic bond
-	-	930 (w)	3,6-Anhydro-galactose [56]
-	-	891 (w)	C-H bending β-galactopyranosyl [56]
622 (sh) 578 (vs) 436 (vs)	615(sh) 572 (vs) 440 (vs)	625(sh) 575 (vs) 436 (vs)	Fe–O-related

**Table 4:** Mössbauer hyperfine fitting parameters for NP M1 and M2. <B_hf_>(σ): average magnetic hyperfine field and standard deviation of magnetic field distribution; IS: isomer shift; ε: quadrupole shift; Γ: Lorentzian line width; I: relative area. Uncertainties in I are less than 2%.

Sample	Site	<B_hf_>(σ) T	IS mm s^−1^	ε mm s^−1^	Γ mm s^−1^	I %
M1	1 (cyan)	43(9)	0.29(1)	−0.01(1)	0.40	75.6
	2 (green)	41(10)	0.56(1)	−0.09(1)	0.30	24.4
M2	1 (cyan)	38(12)	0.28(1)	0.01(1)	0.45	74.2
	2 (green)	37(10)	0.65(1)	0.01	0.35	25.8

**Table 5:** Mössbauer hyperfine fitting parameters for NP M–g, M–p and M–a. <B_hf_>(σ): average magnetic hyperfine field and standard deviation of the magnetic field distribution; IS: isomer shift; ε: quadrupole shift; Γ: Lorentzian line width; I: relative area. Uncertainties in I are less than 2%.

Sample	Site	<B_hf_>(σ) T	IS mm s^−1^	ε mm s^−1^	QS mm s^−1^	Γ mm s^−1^	I %
M–g	1 (cyan)	40(11)	0.28(1)	−0.01(1)	-	0.55	80.2
	2 (green)	38(12)	0.63(1)	−0.01	-	0.35	19.8
M–p	1 (cyan)	29(13)	0.34(1)	−0.01(1)	-	0.45	73.2
	3	-	0.34(1)	-	0.66(1)	0.78(1)	26.8
M–a	1 (cyan)	35(13)	0.27(1)	−0.006 (8)	-	0.55	71.7
	2 (green)	33(13)	0.62(2)	−0.01	-	0.40	28.3

**Table 6:** Mössbauer hyperfine fitting parameters for NP M–pg and M–pa. <B_hf_>(σ): average magnetic hyperfine field and standard deviation of the magnetic field distribution; IS: isomer shift; ε: quadrupole shift; Γ: Lorentzian line width; I: relative area. Uncertainties in I are less than 2%.

Sample	Site	<B_hf_>(σ) T	IS mm s^−1^	ε mm s^−1^	QS mm s^−1^	Γ mm s^−1^	I %
M–pg	1 (cyan)	36(12)	0.33(1)	−0.01(1)	-	0.55	89.4
	3	-	0.32(1)	-	0.68(1)	0.72(3)	10.6
M–pa	1 (cyan)	37(12)	0.33(1)	−0.02(1)	-	0.60	96.0
	3	-	0.33(1)	-	0.62(1)	0.68(4)	4.0

**Table 7:** Zeta potential, pH, mass ratio of citric acid to MNPs, and total Fe concentration for the _CA ferrofluids and _MQ dispersions.

Sample	m_CA_/m_MNP_	ζ mV	pH	C_Fe [ICP]_ mg/mL	C_Fe [LabCal]_ mg/mL
M1_CA	1.2	−18.5 ± 0.5	7.73	2.7	2.6
M2_CA	1.6	−33.8 ± 1.5	7.98	-	2.7
M–g_CA	1.4	−20.0 ± 0.1	7.78	6.3	6.4
M–p_CA	1.3	−32.1 ± 0.8	7.88	6.7	7.3
M–a_CA	1.6	−31.1 ± 1.5	7.41	4.4	4.4
M–pg_CA	1.3	−14.9 ± 1.6	7.54	5.0	5.5
M–pa_CA	1.2	−18.7 ± 0.0	7.16	7.1	7.7
M–p_MQ	-	−36.0 ± 0.1	7.20	-	2.1
M–pa_MQ	-	−37.8 ± 1.9	7.60	-	2.2

**Table 8:** Mean particle sizes (<D_TEM_>) with associated standard deviations from Log-Normal distributions fitted to histograms of ~350 NPs (TEM images). <D_XRD_>: mean particle size determined from XRD data.

Sample	<D_TEM_>/nm	<D_XRD_>/nm
M1_CA	15(4)	16
M2_CA	9(2)	12
M–g_CA	6(3)	11
M–p_CA	7(4)	10
M–a_CA	9(3)	11
M–pg_CA	8(2)	9
M–pa_CA	9(3)	12

**Table 9:** Iron concentration determined using LabCal method, C_Fe[LabCal]_, and using the spontaneous magnetization, C_Fe(MNP)_. The corresponding ratio, C_Fe (MNP)_/C_Fe[LabCal]_, is also shown, as well as the magnetic iron oxide concentration, C_iron oxide_.

Sample	C_Fe[LabCal]_ mg/mL	C_iron oxide_ mg/mL	C_Fe (MNP)_ mg/mL	C_Fe (MNP)_/C_Fe[LabCal]_
M1_CA	4.6	3.61	2.53	55%
M2_CA	2.7	2.05	1.44	57%
M–g_CA	6.4	0.13	0.09	1%
M–p_CA	7.3	4.56	3.19	44%
M–a_CA	4.4	3.44	2.41	54%
M–pg_CA	5.5	2.12	1.48	27%
M–pa_CA	7.7	7.00	4.9	64%
M–p_MQ	2.1	3.01	2.11	100%
M–pa_MQ	2.2	3.07	2.15	100%

**Table 10:** Specific loss power (SLP) determined for the stable MNP suspensions and the corresponding intrinsic loss power (ILP).

Sample	SLP ${\mathrm{Wg}}_{\mathrm{MNP}}^{-1}$	ILP ${\mathrm{nWg}}_{\mathrm{MNP}}^{-1}$
M1_CA	230	6.84
M2_CA	64	2.31
M–g_CA	0	0.0
M–p_CA	79	2.83
M–a_CA	55	1.98
M–pg_CA	78	2.80
M–pa_CA	132	4.74
M–p_MQ	43	1.54
M–pa_MQ	100	3.60

## Data Availability

The data presented in this study are available on request from the corresponding author.
